# Bacterial Colonization and Proliferation in Primary Molars following the Use of the Hall Technique: A Confocal Laser Scanning Microscopy Study

**DOI:** 10.3390/children10030457

**Published:** 2023-02-25

**Authors:** Shlomo Elbahary, Shiran Aharonian, Hanaa Azem, Benjamin Peretz, Olga Mostinski, Sigalit Blumer

**Affiliations:** 1 Department of Endodontology, The Maurice and Gabriela Goldschleger School of Dental Medicine, Tel Aviv University, Tel Aviv, Ramat Aviv 6997801, Israel; 2 Department of Pediatric Dentistry, The Maurice and Gabriela Goldschleger School of Dental Medicine, Tel Aviv University, Tel Aviv, Ramat Aviv 6997801, Israel; 3 Meindentist, 10435 Berlin, Germany

**Keywords:** bacterial load, caries, children, crown, Hall technique, microleakage, restoration

## Abstract

Restorative dentistry aims to create a favorable environment to arrest caries with minimal operative intervention. The Hall technique (HT) involves the seating and cementation of stainless steel crowns (SSC) on primary molars without any tooth preparation, caries removal, or local anesthesia. In this manner, it entombs bacteria and arrests caries’ progress. We compared bacterial distribution and quantity among primary molars affected with caries and restored with SSC using the HT (*n* = 10), the conventional technique (CT; *n* = 10), or not restored at all (control; *n* = 10). The teeth were contaminated with *Enterococcus faecalis* to mimic the clinical situation in the oral cavity and then incubated for 21 days. They were then cut mesiodistally and evaluated with confocal laser scanning microscopy. Total bacterial load (live + dead) in the mesial and distal areas of the crown showed no significant difference between the groups (*p* = 0.711), but there were significantly more dead than live bacteria in the CT and control groups versus the HT group (*p* = 0.0274 and *p* = 0.0483, respectively). Inside the pulp chamber and the crown area, the total bacterial load was significantly higher in the HT compared to the CT group (*p* < 0.001). Significantly more dead than live bacteria were observed in all tooth areas treated with the HT (*p* = 0.0169). Bacterial penetration depth was significantly correlated with bacterial load (*p* = 0.0167). In conclusion, although more bacteria were present in teeth that had undergone the HT versus those treated with the CT, they were mainly unviable. Additionally, the CT and the HT showed a similar performance in terms of marginal leakage, indicating that complete caries removal is not essential to achieve good sealing.

## 1. Introduction

Since their introduction in 1950 [1], preformed metal crowns, commonly called stainless steel crowns (SSC), have been used for restorations of compromised primary dentition. SSCs are recommended by the American Academy of Pediatric Dentistry (AAPD) for treating primary and young permanent teeth with pulp therapy, multi-surface caries, and developmental defects [2]. Although they are considered durable and clinically successful, many clinicians find them difficult to fit due to a lack of patient cooperation, prolonged chair time, and the need to administer local anesthesia to a pediatric patient [3].

The Hall technique (HT) is a method for entombing caries in primary molars where the crown is placed without local anesthesia, caries removal, or tooth preparation [4]. The technique was developed in the 1980s by Dr. Norna Hall and was first reported in the literature in 2006. Dr. Hall worked in an area of Scotland with a high prevalence of caries and low treatment acceptance. To answer to the demand for a treatment that did not involve local anesthesia and had a higher acceptance level among patients and their parents, she gradually adapted conventional crown placement to this technique [5]. In this method, the operator fits the crown of the appropriate size to cover all the crown margins and loads it with glass ionomer cement. Then, the crown is seated over the affected tooth by either the dentist’s finger pressure or the child’s biting force [5]. When the HT is used, as with other non-, or minimally invasive, caries therapy modalities, the superficial plaque layer—the essential layer in the biofilm for caries progression [6]—is sealed along with the carious lesion and cut off from external nutrients in the oral cavity. Subsequently, the plaque biofilm composition changes to less cariogenic flora. Therefore, this technique may arrest or slow caries progression in primary teeth [7]. Case selection is critical for achieving the maximal success rate when applying the HT. Indications include cavitated or non-cavitated teeth with proximal or occlusal lesions in children who cannot accept selective caries removal. Contraindications include teeth with signs or symptoms of irreversible pulpitis, dental infection, clinical or radiographic signs of pulpal exposure, periradicular pathology, and unrestorable teeth with conventional techniques [6].

The HT aims to increase the child’s compliance due to the elimination of local anesthesia, noise, and other possible irritating factors. As utilization of the HT is less traumatic to young children undergoing dental treatments, it may also contribute to the cooperation of children during later dental treatments [8]. As the procedure is simpler and demands less chair time, the HT may also increase the operator’s acceptance.

Use of the HT during the COVID-19 pandemic was advantageous because it does not create an aerosol. Moreover, children’s increased compliance with the technique reduced the burden on clinics [9].

Studies that have evaluated the HT reported high success rates (i.e., no pain or infection) of 99–100% after one year, 93–98% after two years, and 97% after five years [5,7,10]. Despite all the advantages mentioned above, the use of the HT for carious primary molars remains unpopular and controversial. Despite being recognized, it is not used among pediatric dentists worldwide due to a lack of training, its perception as substandard dentistry, and a perceived lack of evidence [11].

One of the most critical factors for the survival of a crown is its marginal seal [12]. The clinically undetectable passage of bacterial toxins and oral fluids may lead to complications and failure within a few years [13]. Therefore, it is essential to assess the influence of different clinical tooth restoration methods with SSCs to understand how these parameters might affect microleakage and, thus, the survival and success rate of the restoration.

Previous studies that evaluated the effectiveness of various restorative techniques comprehensively assessed the microleakage of SSCs using various laboratory and clinical methods. These included the dye penetration test, in which a dye is applied to the tooth, and the depth of dye penetration is measured to evaluate microleakage; high-power electron microscopy to observe the interface between the tooth and the restoration; the fluoride leakage test which uses a solution containing fluoride to measure the amount of fluoride leaking from the restoration; and the radioisotope leakage test, in which radioactive material is placed on the restoration and the amount of radioactivity leaking from the tooth is measured [14]. Several other microscopic techniques have been used to assess the bacterial colonization of dentin, including stereomicroscopy, scanning electron microscopy, and transmission electron microscopy; however, these methods serve mainly for descriptive purposes as they are indirect, not qualitative, and cannot evaluate the viability of bacteria [15,16,17]. Confocal laser scanning microscopy (CLSM) has been used in several studies to evaluate and trace the penetration of bacteria into the dentinal tubules histologically, using dead/live staining. This method was also used to assess bacterial viability inside the dentinal tubules [15,16,17].

No study has histologically traced bacteria’s actual paths and viability following the use of the HT in carious primary teeth using CLSM or other modern histological techniques. Therefore, in this study, we used CLSM and a bacterial viability kit to evaluate and compare bacterial colonization and proliferation in primary molars restored with SSCs that were placed using the conventional technique (CT) and the HT. Our null hypothesis was that there would be no difference between the total bacterial load and the viability between teeth treated using the HT and the CT.

## 2. Materials and Methods

### 2.1. Experimental Groups

Thirty mandibular second primary molars that were freshly extracted for orthodontic reasons were collected for this study. All selected teeth were affected with carious lesions located at the proximal and occlusal areas, and graded six according to the International Caries Detection and Assessment System (ICDAS) [18]. Radiologically there were no signs of pulp penetration, and the strip of 1 mm of sound dentin was present between the carious lesion and the pulp. The teeth were stored in sterile saline until the experiments.

The teeth were divided into three groups:

Group 1 (*n* = 10): the teeth were restored using the conventional technique (CT). The mesiodistal dimension of the teeth was determined using a periodontal probe before the most appropriate SSC was selected. The occlusal surface was reduced by 1.0 to 1.5 mm using a K2 diamond bur (Strauss & Co, Ra’anana, Israel) with a high-speed handpiece. The caries was removed with a large, round no. 8 carbide bur (Komet Dental, Lemgo, Germany) using a low-speed handpiece. The proximal reduction was accomplished with a thin, tapered diamond bur (E1, Strauss & Co). All line angles were rounded using the side of the bur. The occluso-buccal and occluso-lingual line angles were rounded by holding the bur at a 30- to 45-degree angle to the occlusal surface and sweeping it in a mesiodistal direction. A pre-contoured and pretrimmed SSC (3M ESPE Stainless Steel Crowns, Minneapolis, MN, USA) was fitted and crimped using 800-417 pliers (3M ESPE Stainless Steel Crowns). A crown was placed on the prepared teeth using finger pressure and cemented using glass ionomer cement. (FUJI1 GC Europe, Leuven, Belgium), followed by bacterial contamination.

Group 2 (*n* = 10): The HT was performed without caries removal. The teeth were not prepared and the SSCs were not pre-crimped. The smallest possible pre-contoured and pretrimmed SSC (3M ESPE Stainless Steel Crowns) covering all crown margins was selected and fitted on each tooth according to its dimensions. The crown was placed on the prepared teeth using finger pressure and cemented using glass ionomer cement (FUJI1 GC Europe), followed by bacterial contamination.

Group 3 (*n* = 10) served as control: caries was removed with a large, round no. 8 carbide bur (Komet Dental) using a low-speed handpiece without SSC placement followed by bacterial contamination.

### 2.2. Experimental Model

**Figure 1:**
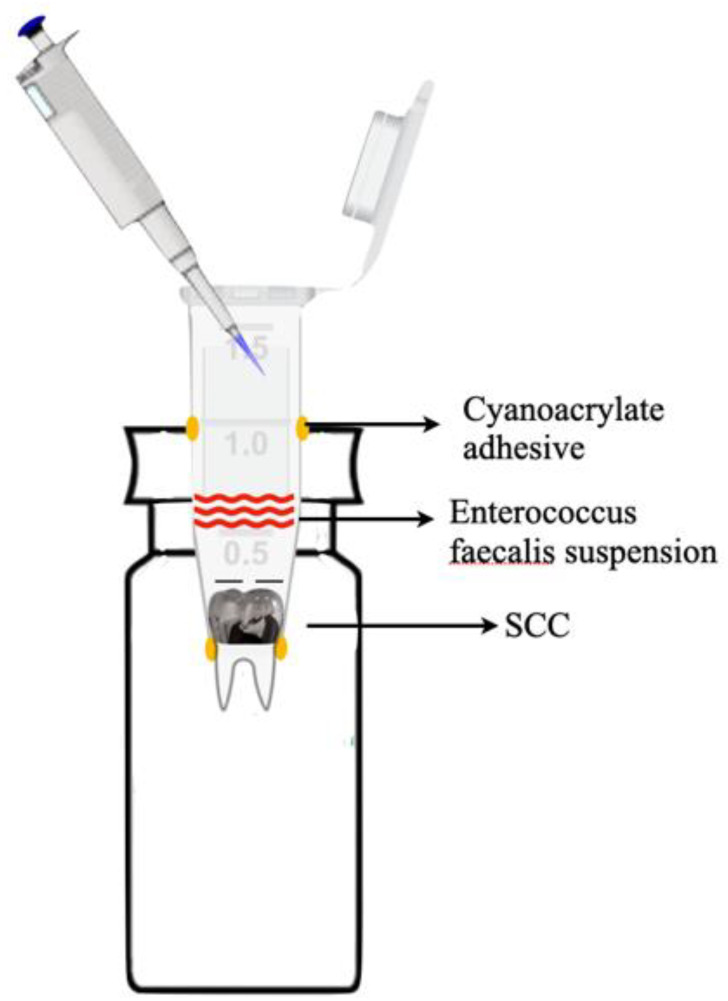
Experimental model. Each tooth was placed in a 1.5 mL Eppendorf plastic tube and then inserted into a 20 mL disposable glass scintillation vial through the opening of the rubber cap so that the plastic tube fitted tightly inside the glass vial. The junctions between the teeth, the Eppendorf tube, and the rubber cap were sealed with a cyanoacrylate adhesive. The system was then sterilized overnight using ethylene oxide gas.

All roots were mounted using a model as described previously [15,16,17]. Briefly, each tooth was placed in a 1.5 mL Eppendorf plastic tube (Sigma-Aldrich Co., St. Louis, MO, USA) and then inserted into a 20 mL disposable glass scintillation vial (Sigma-Aldrich Co) through the opening of the rubber cap, so that the plastic tube fitted tightly inside the glass vial. The junctions between the teeth, the Eppendorf tube, and the rubber cap were sealed with a cyanoacrylate adhesive (Krazy Glue, Colombus, OH, USA) (Figure 1). The system was then sterilized overnight using an ethylene oxide gas [19].

### 2.3. Simulation of Enterococcus faecalis Bacterial Infection

A growth medium for streptomycin-resistant T2-strain *Enterococcus faecalis* (*E. faecalis*) (ATCC^®^ 29212™, ATCC, Manassas, VA, USA) was prepared and autoclaved. To prevent contamination by additional bacterial species, 0.5 mg/mL streptomycin sulfate (Sigma-Aldrich Co.) was added, as *E. faecalis* is resistant to this concentration of streptomycin sulfate. Each tooth specimen was filled from the coronal part of the root canal with the freshly prepared bacterial suspension and then incubated at 37 °C and 100% humidity. The bacterial suspension was replaced with a fresh preparation every 24 h for 21 days.

### 2.4. Preparation of Samples for Evaluation

**Figure 2:**
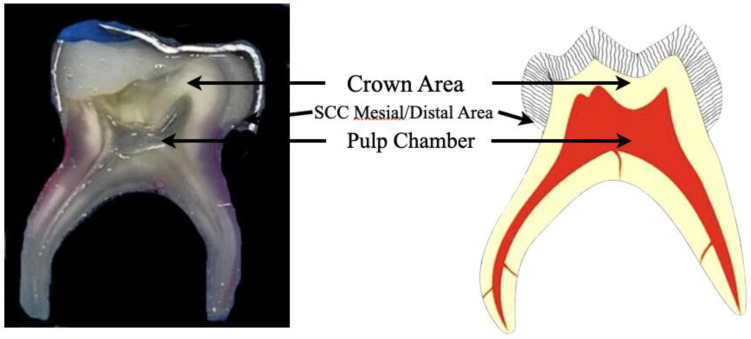
Preparation of teeth for confocal laser scanning microscopy. Mesiodistal coronal plane cuts were performed, and the teeth were stained with a LIVE/DEAD BacLight Bacterial Viability Kit.

After 21 days of incubation, the tooth specimens were embedded in a self-cure acrylic repair material (Triad VLC resin; Dentsply, Int., York, PA, USA), and 0.2 mm mesiodistal coronal plane cuts were performed through the SCC and root area of each specimen with a diamond saw rotating at 500 rpm (Isomet, Buehler Ltd., Lake Bluff, IL, USA), under water cooling (Figure 2). The samples were stained using a LIVE/DEAD BacLight Bacterial Viability Kit L-7012 (Molecular Probes, Eugene, OR, USA) containing separate vials of the two-component dyes (SYTO 9 and propidium iodide in 1:1 mixture) for the staining of the biofilm. These dyes’ excitation/emission maxima are 480–500 nm for the SYTO 9 stain and 490–635 nm for propidium iodide.

### 2.5. Confocal Laser Scanning Microscopy

**Figure 3:**
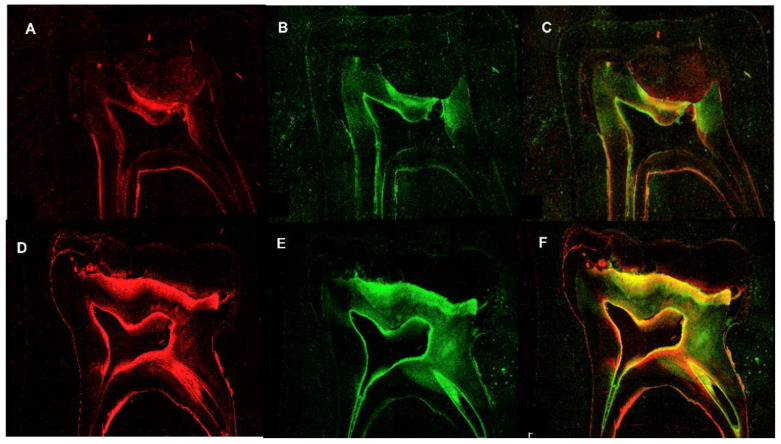
(**A**–**F**): Confocal laser scanning microscopy images of bacterial colonization. The infected dentin was stained with a LIVE (green)/DEAD (red) BacLight Bacterial Viability Kit and analyzed using the LAS AF software. (**A**–**C**) Conventional technique, (**D**–**F**) Hall technique.

Immediately after the staining procedure, fluorescence from the stained bacteria was observed under a confocal laser scanning microscope (CLSM; Leica TCS SP5, Leica Microsystems CMS GmbH, Wetzlar, Germany). Single-channel imaging and simultaneous dual-channel imaging were used to display green and red fluorescence (Figure 3).

The mesial, distal, pulp chamber, and crown areas of the specimens were evaluated by the software as follows:

1. The extent of fluorescent staining within the evaluated areas was calculated.
2. The vitality of the colonized bacteria was calculated as the proportion of live vs. dead bacteria.
3. The distance between the bacterial load in the crown area and the pulp chamber was measured.

### 2.6. Statistical Analysis

The variables are presented as the median and interquartile range and compared among all groups with Kruskal–Wallis tests. Post hoc Nemenyi tests were conducted on variables for which differences were found. Multivariate generalized linear models were used for comparing bacteria by status (alive versus dead) and group. A Spearman correlation assessed the association between distance and bacteria in the pulp chamber.

## 3. Results

Bacterial colonization of the infected teeth in each experimental group is shown in Figure 3.

**Figure 4:**
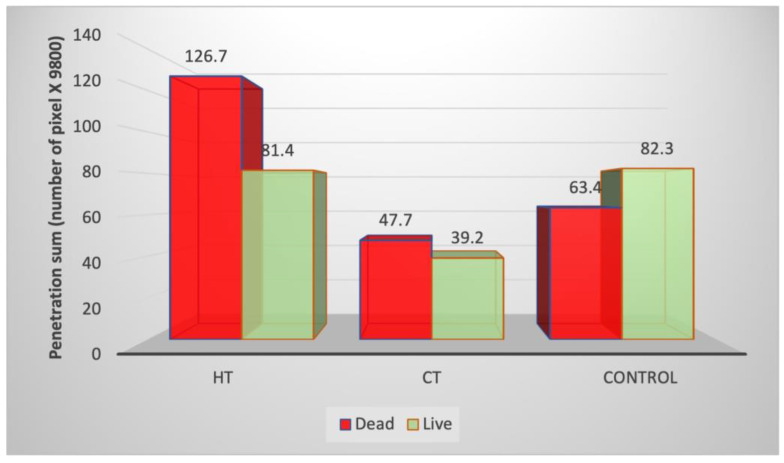
Total bacterial load of live and dead bacteria in the experimental groups. Abbreviations: CT, conventional technique; HT, Hall technique.

The quantification of bacterial colonization in the experimental groups is depicted in Figure 4. There was a significantly higher total bacterial load in the HT group compared with the CT group (*p* < 0.001) and a higher total bacterial load in the control group compared with CT (*p* = 0.0053). There were significantly more live bacteria in the HT compared with the CT group (*p* = 0.04). In the HT group, there were significantly more dead than live bacteria in all areas (*p* = 0.0169).

**Figure 5:**
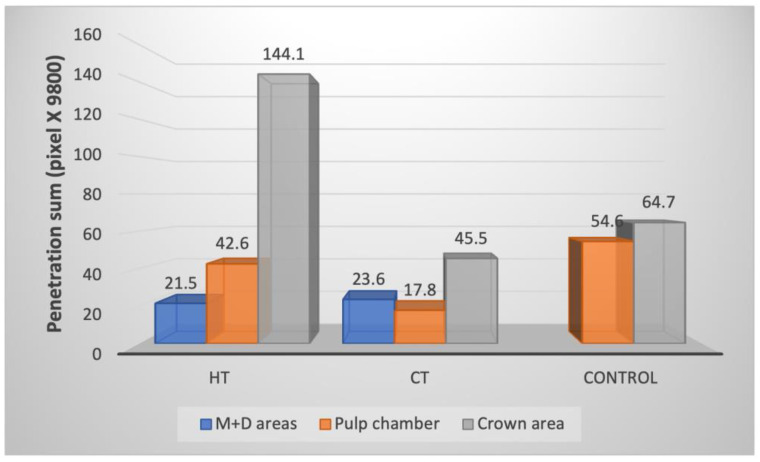
Comparison of total bacterial distribution among the different parts of the tooth and by experimental group. Abbreviations: CT, conventional technique; HT, Hall technique; M + D, mesial and distal.

Analysis of bacterial load by tooth anatomy (Figure 5) showed no significant differences between groups in the total (live + dead) bacterial load at the mesial and distal areas of the SSC (*p* = 0.711). There were significantly more dead than live bacteria in the CT group (*p* = 0.0274).

Significantly more bacteria (live and dead) were observed inside the pulp chamber of the HT group compared to the CT group (*p* < 0.001). There were significantly more dead bacteria than live in the pulp chamber of the HT group (*p* = 0.0099).

**Figure 6:**
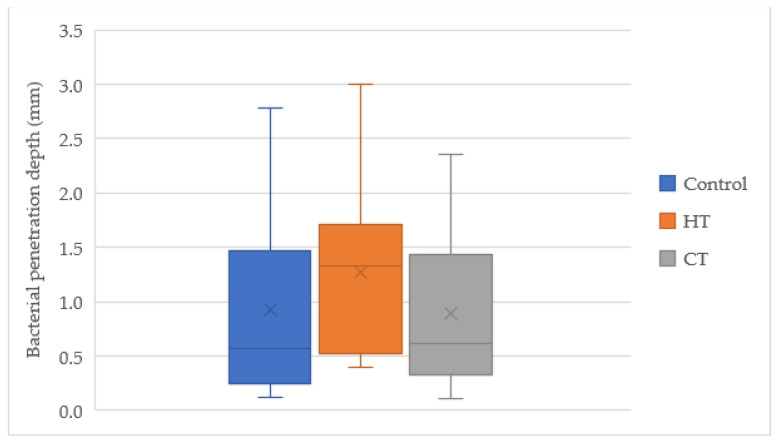
Distance (mm) between the bacterial load in the crown area and the pulp chamber. *p* = 0.0167. Abbreviations: CT, conventional technique HT, Hall technique; M + D, mesial and distal.

There was a significant association between the bacterial load in the crown area and the penetration distance to the pulp chamber (*p* = 0.0167) (Figure 6).

## 4. Discussion

Dental caries is a multifactorial disease that results from an ecologic shift within the dental biofilm to acidogenic and aciduric bacterial species, influenced by host factors such as saliva quality and quantity, diet, and others. The loss of equilibrium between demineralization and remineralization processes leads, given enough time, to tooth substance loss and clinically visible decay.

Bacterial colonization of dentin tubules is an active process mediated by cell division and availability of nutrients [20]; therefore, the main goal of restorative treatment is to prevent the invasion of bacteria and their by-products in the oral cavity. The paradigm of early accepted approaches, where carious tissue was removed and restoration was placed, has shifted to more biological methods with minimal invasive interventions [10]. This approach focuses on arresting lesions and disturbing or modifying plaque by placing an adequate restoration that seals the cavity without jeopardizing pulpal vitality, rather than removing caries [21].

An ideal restoration should prevent the subsequent leakage of bacterial by-products into the pulp and peri-radicular tissues [22]. Prior studies have shown that the number of viable bacteria in the carious dentin beneath a restoration decreases over time and ‘‘dries out’’, suggesting lesion arrest. This may be explained by the restriction of an exogenous nutrient supply, which isolates the caries process from the oral cavity, and the observed shift toward less cariogenic microflora [23,24].

To understand the mechanism of bacterial behavior following the restorative process, an experimental model should be capable of assessing the effectiveness of sealing with the chosen restorative method, and tracking and quantifying the routes and extent of bacterial colonization. *E. faecalis* was chosen because this species exist in the normal oral flora in humans, is frequently found in mixed infections with other aerobes and facultative anaerobes, and does not form endospores [25,26]. *E. faecalis* also plays an essential role in bacterial biofilm formation and therefore is considered an appropriate model for testing novel treatments [14,27,28,29,30].

In the current study, the actual routes of microbial colonization were histologically traced using CLSM, and positive and negative histological controls were used to confirm the adequacy of the experimental model. CLSM can be a valuable tool for dental research. It provides high-resolution, three-dimensional images of tooth structure and restorative materials, which can help researchers better understand the structure–function relationship in teeth and how various restorative materials interact with the tooth structure. CLSM has been used in multiple dental research studies, including caries detection, microleakage evaluation, and evaluation of the bonding effectiveness of dental adhesives. Additionally, CLSM can visualize living cells and tissues in real time, providing valuable information for research on biological processes in the oral cavity, such as tooth development, wound healing, and disease progression. Overall, CLSM has the potential to enhance the understanding of dental biology and material science significantly, making it a valuable tool for dental research [7,8,9,10,11,12,13,14,15,16,17,18,19]. In our study, CLSM provided direct and quantifiable information about the presence and distribution of live and dead *E. faecalis* bacteria inside the dentinal tubules [31].

We observed microleakage in all groups, but no significant differences were observed between groups in the total (live + dead) bacterial load at the mesial and distal areas of the SSC. Furthermore, the viability of the colonized bacteria was affected by the way the crown was treated: more live and dead bacteria were found in the HT in comparison with the CT group, and there was a significant association between the bacterial load and the penetration distance, probably because the degree of microleakage is greater in dentin than in enamel [32]. In addition, cavity preparations on progressively decayed teeth may result in stressed pulp [33]. Further placement of restorations with inadequate seals could worsen the tooth condition leading to ‘major’ failures.

Our finding that the bacterial load following HT was higher than the bacterial load after CT is in concordance with the findings of other studies [34], as more microorganisms were detected in teeth submitted to partial caries removal compared with complete caries removal. However, the number of viable bacteria and their pathogenicity decreases over time [35]. In the current study, the observations were limited to one month, and a more extended follow-up period is needed for a more accurate evaluation.

Luting materials and the technique significantly affected SSC microleakage. Glass ionomers have been shown to be relatively insoluble, biocompatible, and bacteriostatic, minimizing microleakage [36,37]. Therefore, they can be considered a contributing factor to defense against bacteria and bacterial leakage [38]. Cement thickness does not negatively affect the marginal seal [39], but this variable deserves further research. Occlusal load stress, which occurs typically under in vivo conditions, is another variable that was not tested in the current study and should be considered when the marginal leakage of cemented crowns is tested. Restorative dentistry aims to create a favorable environment to arrest caries with minimal operative intervention. Our findings indicate that removing the excavation of all carious tooth material from cavitated lesions may not be essential to controlling caries progression and management.

The present study is a part of increasing evidence-based findings on pragmatic clinical trials that shows HT is a fast and non-invasive procedure that does not require tooth preparation and reports a high success rate for treating carious, vital, symptomless primary molar teeth [5,40,41,42,43]. This technique is advantageous for behavior management in children with a history of negative dental experience, fear, and dental anxiety. Treatment should be chosen based on a patient’s risk levels, age, and compliance. Proper case selection will confer successful outcomes of sealing carious primary molars. The HT is designed to help children cooperate and be more comfortable during treatment without general anesthesia. However, it is essential to note that the HT may not be appropriate for every child and every situation.

As the experimental conditions in the current study did not fully mimic the clinical situation, the extent of microleakage following the utilization of the HT should be established in long-term clinical trials.

## 5. Conclusions

This work used different techniques to shed light on the actual paths of bacterial infection in dental tubules during SSC placement on primary molars. More bacteria were present in teeth that had undergone the HT compared to the CT, although they were mainly unviable. Additionally, CT and HT showed a similar performance in terms of marginal leakage, indicating that complete caries removal is not essential to achieve good sealing.

## Data Availability

The data supporting the results are available from the corresponding authors upon reasonable request.
